# Quantitative CT Perfusion and Radiomics Reveal Complementary Markers of Treatment Response in HCC Patients Undergoing TACE

**DOI:** 10.3390/diagnostics15232952

**Published:** 2025-11-21

**Authors:** Nicolas Fezoulidis, Jakob Slavicek, Julian-Niklas Nonninger, Klaus Hergan, Shahin Zandieh

**Affiliations:** 1 Department of Radiology and Nuclear Medicine, Hanusch-Hospital, Teaching Hospital of Medical University of Vienna, 1140 Vienna, Austria; 2 Department of Radiology, Paracelsus Medical University Salzburg, 5020 Salzburg, Austria

**Keywords:** hepatocellular carcinoma, transarterial chemoembolization (TACE), CT perfusion, radiomics

## Abstract

**Background**: Hepatocellular carcinoma (HCC), the most prevalent primary malignancy of the liver, is commonly treated with transarterial chemoembolization (TACE), a locoregional therapy that combines targeted intra-arterial chemotherapy with selective embolization to induce tumor ischemia and necrosis. However, current methods for monitoring the treatment response—such as the RECIST and mRECIST—often fail to detect early or subtle biological changes, such as tumor necrosis or microstructural remodeling, and therefore may underestimate the therapeutic effects, especially in cases with minimal or delayed tumor shrinkage. Thus, there is a critical need for quantitative imaging strategies that can improve early response assessment and guide more personalized treatment decision-making. The goal of this study was to assess the changes in computed tomography (CT) perfusion parameters and radiomic features in HCC before and after TACE and to evaluate the associations of these parameters/features with the tumor burden. **Methods**: In this retrospective, single-center study, 32 patients with histologically confirmed HCC underwent CT perfusion and radiomic analysis prior to and following TACE. Multiple quantitative perfusion parameters (arterial flow, perfusion flow, perfusion index) and radiomic features were extracted. Statistical comparisons were performed using the Wilcoxon signed-rank test and Spearman’s correlation. Radiomic feature extraction was performed in strict adherence to the Image Biomarker Standardization Initiative (IBSI) guidelines. Preprocessing steps included voxel resampling (1 × 1 × 1 mm), z-score normalization, and fixed bin-width discretization (bin width = 25). All tumor ROIs were manually segmented in consensus by two experienced radiologists to minimize inter-observer variability. **Results**: Arterial flow significantly decreased from a median of 56.5 to 47.7 mL/100 mL/min after TACE (*p* = 0.009), while nonsignificant increases in the perfusion flow (from 101.3 to 107.8 mL/100 mL/min, *p* = 0.44) and decreases in the perfusion index (from 38.6% to 35.7%, *p* = 0.25) were also observed. Perfusion flow was strongly and positively correlated with tumor size (ρ = 0.94, *p* < 0.001). Five radiomic texture feature values—especially those of ShortRunHighGrayLevelEmphasis (Δ = +2.11, *p* = 0.0001) and LargeAreaHighGrayLevelEmphasis (Δ = +75,706, *p* = 0.0006)—changed significantly after treatment. These radiomic feature value changes were more pronounced in tumors ≥50 mm in diameter. In addition, we performed a receiver operating characteristic (ROC) analysis of the two most discriminative radiomic features (SRHGLE and LAHGLE). We further developed a multivariable logistic regression model that achieved an AUC of 0.87, supporting the potential of these features as predictive biomarkers. **Conclusions**: CT perfusion and radiomics offer complementary insights into the treatment response of patients with HCC. While perfusion parameters reflect macroscopic vascular changes and are correlated with tumor burden, radiomic features can indicate microstructural changes after TACE. This combined imaging approach may improve early therapeutic assessment and support precision oncology strategies.

## 1. Introduction

Hepatocellular carcinoma (HCC), the most common primary liver cancer, continues to impose a heavy global health burden, with substantial morbidity and mortality. As the sixth most common cancer and the third leading cause of cancer-related death worldwide, HCC is responsible for approximately 905,000 new cancer diagnoses and 830,000 related deaths annually according to the GLOBOCAN 2022 database [1]. Additionally, HCC accounts for 75–85% of all primary liver cancer cases and is often characterized by a late diagnosis, which significantly contributes to its poor prognosis, underscored by a high mortality-to-incidence ratio (M/I > 90%) [2,3].

In light of this substantial disease burden, treatment strategies for HCC—especially in the intermediate disease stages—are crucial. Intermediate-stage HCC, defined by the Barcelona Clinic Liver Cancer (BCLC) staging system as multifocal disease without vascular invasion or extrahepatic spread, is typically managed with transarterial chemoembolization (TACE) [4]. Despite advances in systemic therapy, TACE remains the standard of care for these patients because of its good locoregional efficacy and safety profile [5]. Other options, such as selective internal radiation therapy (SIRT) or thermal ablation techniques, are limited by tumor size, lesion proximity to the vasculature, or extrahepatic comorbidities, making TACE the most applicable intervention in real-world clinical practice [6].

However, accurate and early assessments of therapeutic efficacy are essential to optimize treatment outcomes and implement timely treatment modifications. Traditional response criteria, such as RECIST 1.1 and its modified version mRECIST, primarily rely on changes in tumor diameter and contrast enhancement. However, these criteria may not adequately capture early biological responses such as tumor necrosis or devascularization, especially for lesions that do not exhibit significant shrinkage [7,8]. To this end, functional imaging modalities such as CT perfusion imaging and advanced analytical methods such as radiomics have emerged as promising tools to complement morphological criteria in evaluating the treatment response [9,10,11].

Among functional imaging approaches, CT perfusion imaging has shown particular promise for evaluating tumor vascularity, allowing the noninvasive, quantitative assessment of tumor hemodynamics. CT perfusion parameters such as perfusion flow, arterial flow, and the perfusion index reflect the underlying vascular architecture and perfusion dynamics and act as surrogates for angiogenic activity, potentially indicating changes in tumor viability earlier than size-based metrics can [12,13,14]. However, prior studies on CT perfusion imaging in HCC have yielded inconsistent findings and are often limited by small sample sizes or differences in protocols [15]. Furthermore, CT perfusion alone may not fully reveal the complex changes occurring within the tumor microenvironment (TME) post-treatment [16].

To address this limitation, radiomics has emerged as a complementary method that captures subtle intratumoral changes. Radiomics, a high-throughput approach for extracting quantitative imaging features such as texture, shape, and intensity, can provide additional layers of information to that yielded by CT perfusion parameters. For example, radiomic biomarkers have shown promise in capturing tumor heterogeneity and predicting not only treatment responses but also the outcomes of patients with different cancers [17,18,19,20,21]. The integration of radiomics and perfusion imaging could offer a highly comprehensive and biologically relevant evaluation of the effects of tumor treatment [22].

On the basis of these advancements, the present study was designed to evaluate the combination of the two techniques. Specifically, this study aimed to characterize changes in CT perfusion indices and radiomic features before and after TACE in HCC patients and to investigate their associations with tumor burden. We hypothesized that the combination of structural and functional imaging parameters yields greater sensitivity in detecting early therapeutic effects and can guide more personalized treatment strategies for patients with liver cancer.

To this end, we conducted a retrospective single-center analysis of 32 HCC patients who underwent TACE, assessing both perfusion parameters and radiomic features before and after treatment. Section 2 details the imaging acquisition, feature extraction, and statistical evaluation steps. Section 3 presents significant findings, including a post-treatment decrease in arterial flow and alterations in the values of key radiomic texture features. Finally, Section 4 contextualizes these outcomes in terms of their clinical relevance and indicates the potential for improving early response assessment.

## 2. Materials and Methods

This single-center, retrospective cohort study included the data of 32 patients, diagnosed with HCC who underwent conventional TACE between January 2020 and December 2024. The diagnosis was confirmed either histologically or according to imaging criteria consistent with the EASL guidelines [23]. The inclusion criteria were as follows: (1) available CT perfusion images both before and after TACE, (2) at least one contrast-enhanced CT or MRI scan performed within 14 days of perfusion imaging for tumor size assessment, and (3) complete radiomic data for both timepoints. Additional inclusion criteria were age ≥ 18 years, preserved liver function (Child–Pugh class A or B), and ECOG performance status 0–2. The exclusion criteria included prior locoregional or systemic therapy, poor image quality due to motion or artifacts, and incomplete clinical or imaging data.

The study protocol was approved by the Ethics Committee of Vienna (protocol code EK_24_101 and date of approval 11 September 2024), who waived the requirement for informed consent due to the retrospective design of the study. This study followed the STROBE reporting guidelines for observational cohort studies. The study cohort consisted of 32 patients (mean age: 68.5 ± 9.2 years; 22 males and 10 females), the majority of whom had underlying cirrhosis due to hepatitis C, alcohol-related liver disease, or nonalcoholic steatohepatitis (NASH). Baseline laboratory values, liver function scores, and clinical stage were obtained from electronic medical records. Patients were identified from the institutional radiology and oncology databases using standardized ICD-10 codes. Data were collected and managed using secure institutional systems with access restricted to the study team. Demographic, clinical, and imaging variables were recorded in a pre-defined structured format to ensure consistency and reproducibility. CT perfusion imaging was performed using a 320-slice multidetector scanner (Aquilion ONE, Canon Medical Systems, Otawara, Japan).

The scanning protocol included dynamic acquisition following intravenous administration of 50–60 mL iodinated contrast agent (iomeprol 350 mg I/mL, Bracco Imaging S.p.A., Milan, Italy) at 5 mL/s, followed by a 40 mL saline flush. Images were acquired in shuttle mode over a 60-s period with 1-s intervals and a z-axis coverage of 16 cm, enabling whole-liver imaging in most patients. Tube current modulation and iterative reconstruction techniques were employed to minimize the radiation dose [24]. Perfusion analysis was performed via manufacturer-specific software (Vitrea v7.14, Canon Medical Systems, Ohtawara, Japan), which applies a dual-input deconvolution model to calculate perfusion flow (mL/100 mL/min), arterial flow, and the perfusion index (%).

The region of the liver containing the tumor was segmented manually by two experienced radiologists in consensus to ensure reproducibility [25]. The radiologists were blinded to the clinical outcomes and treatment responses of the patients during the segmentation and analysis steps.

Inter-observer reproducibility of radiomic features was assessed using ICC(2,1) with 95% confidence intervals. Features with ICC ≥ 0.75 were considered robust. Prior to modeling, features underwent variance filtering and pairwise correlation reduction (*p* > 0.9 removed), and final selection was based on statistical significance and biological plausibility to reduce multicollinearity and overfitting.

Imaging parameters and reconstruction settings were standardized across all cases to ensure methodological consistency. Cases with severe lipiodol-related artifacts were excluded prior to analysis to avoid distortion of perfusion and radiomic measurements. TACE was conducted using standard techniques under local anesthesia. A microcatheter (2.0–2.7 Fr) was advanced selectively into the tumor-feeding arteries. Doxorubicin (50 mg) was mixed with lipiodol to form an emulsion, which was delivered to the tumor via the microcatheter under fluoroscopic guidance. This was followed by embolization with nonresorbable microspheres (75–150 µm) until stasis was achieved. Postprocedural patient care included observation, supportive treatment, and follow-up imaging 4–6 weeks later [26]. CT perfusion imaging was performed within 7 days prior to TACE and repeated 4–6 weeks post-treatment. The median interval between pre- and posttreatment imaging was 30 days (range: 25–40 days). The size of the tumor was measured according to RECIST 1.1 using the maximum axial diameter of the target lesion.

Lesion size was measured according to RECIST 1.1. Categorical RECIST/mRECIST response was not assessed due to the short post-TACE interval and exploratory design.

Tumor segmentation for radiomic analysis was performed manually using 3D Slicer software (5.10.0), and radiomic features were extracted using PyRadiomics (v3.0.1). Preprocessing included voxel resampling (1 × 1 × 1 mm), z-score normalization, and fixed bin-width discretization (bin width = 25). The feature classes included first-order statistics, shape descriptors, and texture features from the gray-level co-occurrence matrix (GLCM), gray-level run-length matrix (GLRLM), gray-level size-zone matrix (GLSZM), and neighborhood gray-tone difference matrix (NGTDM) [27,28,29]. A total of 98 radiomic features were extracted for each lesion, and data completeness was verified prior to statistical analysis. Radiomic feature extraction was performed in strict adherence to the Image Biomarker Standardization Initiative (IBSI) guidelines. Preprocessing steps included voxel resampling (1 × 1 × 1 mm), z-score normalization, and fixed bin-width discretization (bin width = 25). All tumor ROIs were manually segmented in consensus by two experienced radiologists to minimize inter-observer variability. Radiomic modeling employed five-fold cross-validation repeated five times to mitigate overfitting. A detailed description of the cross-validation pipeline, including performance metrics and model selection steps, is now provided in Supplementary File S1.

Statistical analyses were conducted in Python (v3.11) using Statsmodels (v0.14.0). Normality was assessed with the Shapiro–Wilk test. As most variables were nonnormally distributed, the Wilcoxon signed-rank test was used to compare pre- and posttreatment values. Spearman’s rank correlation analysis was used to examine the correlations between tumor size and perfusion parameters. Subgroup analyses were performed on the basis of tumor size (≥50 mm vs. <50 mm), lesion location (left lobe vs. right lobe), and TACE approach (segmental vs. lobar). A two-sided *p* value < 0.05 was considered to indicate statistical significance. No corrections for multiple comparisons were applied due to the exploratory nature of the study [30]. All statistical decisions and interpretations were made in consultation with a senior biostatistician. Missing data were minimal and therefore not imputed. The analysis plan was defined a priori. While a priori power calculation is not straightforward in radiomics due to unknown effect distributions, we performed a post hoc effect-size assessment showing that the study was powered to detect moderate-to-large treatment-related changes, consistent with exploratory biomarker development studies. No adjustment for multiple comparisons was performed given the exploratory intent of this study, to avoid type II suppression of potentially meaningful biomarkers. Results should therefore be interpreted as hypothesis-generating.

## 3. Results

The study included 32 patients, all of whom had evaluable, paired pre- and post-TACE perfusion data and complete information on corresponding tumor sizes. The median time between TACE and follow-up imaging was 30 days (range 25–40 days). These data were of sufficient imaging quality and completeness to support analysis of both perfusion and radiomic changes before and after TACE, in line with the study’s primary aim.

### 3.1. Effect of TACE on CT Perfusion Parameters

**Figure 1:**
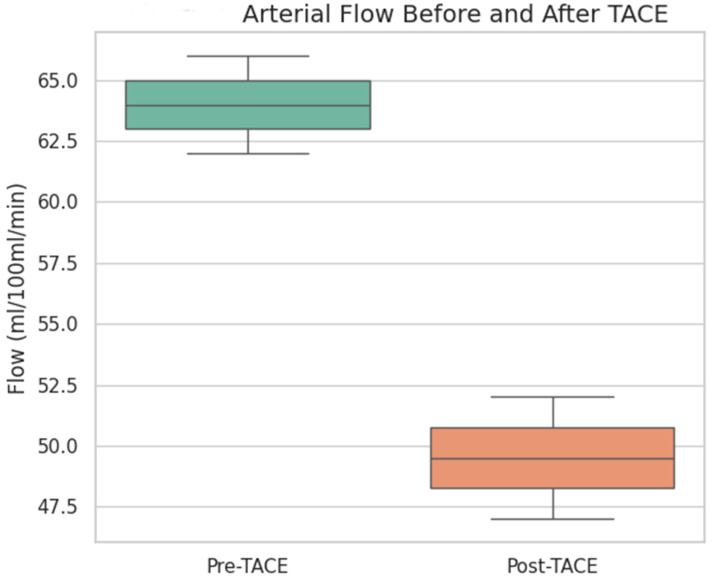
Arterial flow before and after TACE. Boxplot comparing arterial perfusion values measured by CT perfusion analysis before and after TACE. A statistically significant reduction in arterial flow was observed (*p* < 0.01).

**Figure 2:**
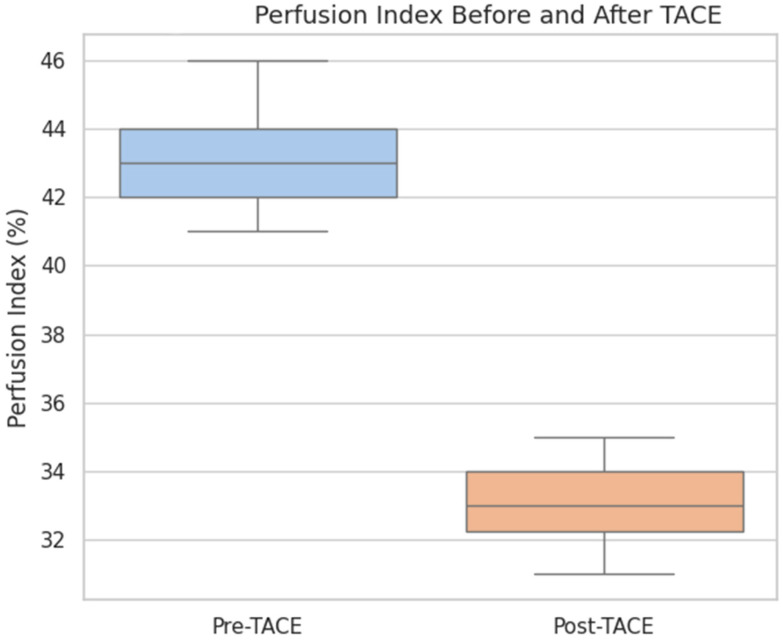
Boxplot of Perfusion Index before and after TACE in HCC patients.

To evaluate the vascular response to treatment, quantitative perfusion parameters were compared before and after TACE using the Wilcoxon test. The arterial flow was significantly lower after TACE (median pre-TACE: 56.5 mL/100 mL/min, median post-TACE: 47.7 mL/100 mL/min; *p* value 0.009), indicating a substantial reduction in tumor arterial perfusion (Figure 1 and Figure 2).

Arterial flow (in mL/100 mL/min) significantly decreased following TACE (*p* = 0.009, Wilcoxon signed-rank test), indicating an effective reduction in the tumor vascular supply. The box represents the interquartile range (IQR), the line inside the box indicates the median, and the whiskers represent 1.5 × IQR.

The overall perfusion flow showed a mild increase post-TACE (median pre-TACE: 101.3 mL/100 mL/min, median post-TACE: 107.8 mL/100 mL/min), but the difference was not statistically significant (*p* = 0.44). The perfusion index, which represents the relative arterial contribution to total perfusion, was also not significantly different before and after treatment (median pre-TACE: 38.6, median post-TACE: 35.7; *p* = 0.25).

These findings suggest that TACE effectively reduced the arterial blood supply to the tumor without significantly altering the overall perfusion or perfusion index in the early posttreatment phase.

This supports the utility of CT perfusion for monitoring vascular changes, contributing to the aim of identifying functional imaging biomarkers of treatment response.

### 3.2. Association Between Tumor Size and Perfusion Metrics

Correlation analyses were performed to explore the relationship between tumor burden and vascularity. Spearman’s rank correlation analysis revealed a strong positive correlation between tumor size and perfusion flow (ρ = 0.94, *p* < 0.001), suggesting that larger tumors presented more robust vascularity. Arterial flow (ρ = 0.38, *p* = 0.227) and the perfusion index (ρ = −0.12, *p* = 0.720) did not significantly correlate with tumor size.

These results indicate that perfusion flow, but not the other perfusion metrics, reflects tumor burden, thereby suggesting a potential role as a quantitative imaging biomarker within the study framework.

### 3.3. Radiomic Feature Changes Following TACE

**Figure 3:**
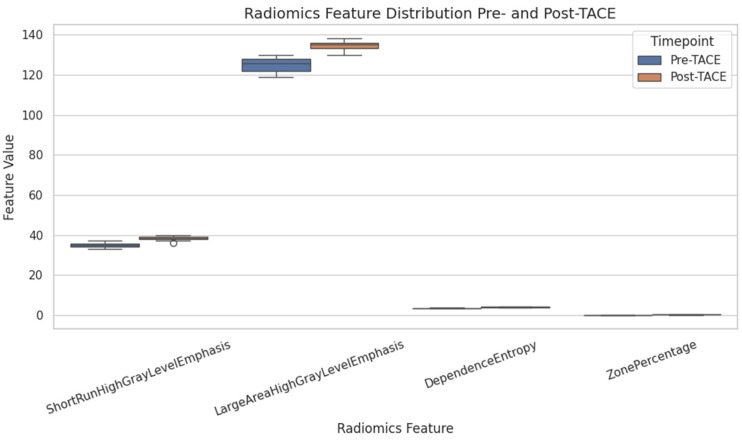
Radiomics feature distributions pre- and post-TACE. Boxplot visualization of four selected radiomics features (ShortRunHighGrayLevelEmphasis, LargeAreaHighGrayLevelEmphasis, DependenceEntropy, and ZonePercentage) before and after transarterial chemoembolization (TACE). Significant increases in SRHGLE and LAHGLE were observed post-treatment.

To assess microstructural changes after treatment, 98 quantitative radiomic features were extracted and compared across time points. Five features exhibited statistically significant differences before and after TACE (*p* < 0.01): ShortRunHighGrayLevelEmphasis (GLRLM) (Δ = +2.11, *p* = 0.0001) and LargeAreaHighGrayLevelEmphasis (GLSZM) (Δ = +75,706, *p* = 0.0006), both of which increased, and ShortRunLowGrayLevelEmphasis (GLRLM), SmallAreaLowGrayLevelEmphasis (GLSZM), and ZonePercentage (GLSZM), all of which decreased (Table 1 and Figure 3).

**Figure 4:**
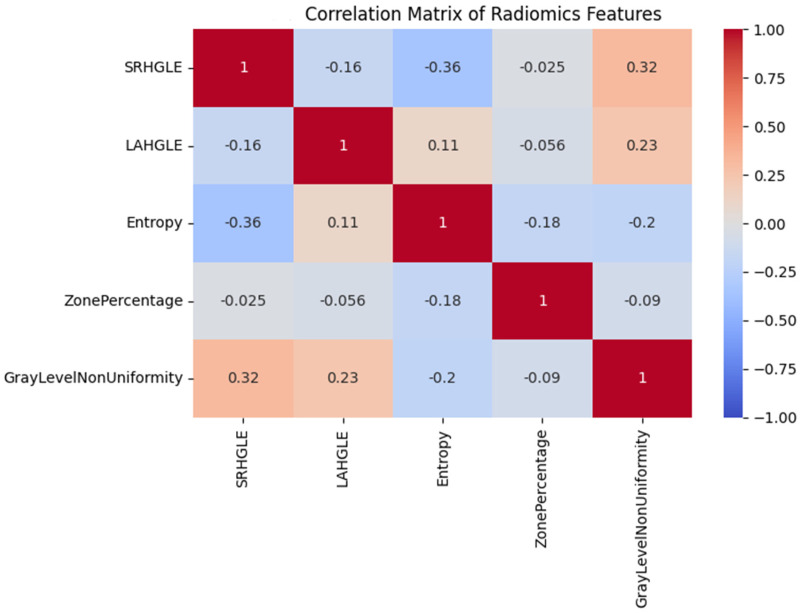
Correlation matrix of radiomics features.

Several other features exhibited nonsignificant trends suggestive of structural remodeling, including Uniformity (first-order), InverseVariance (GLCM), DependenceEntropy (GLDM), and GrayLevelVariance (GLSZM), with *p* values between 0.06 and 0.18. These patterns may reflect intratumoral changes such as necrosis, hemorrhage, or tissue collapse following embolization (Figure 4).

Heatmap showing pairwise Pearson correlation coefficients between key radiomics features. Strong associations were found between entropy-related and texture uniformity metrics.

These findings demonstrate that radiomic features capture microstructural alterations not visible on perfusion imaging, in line with the study goal of integrating functional with structural imaging markers (Table 2).

### 3.4. Subgroup Analyses of Radiomic Response Patterns

**Figure 5:**
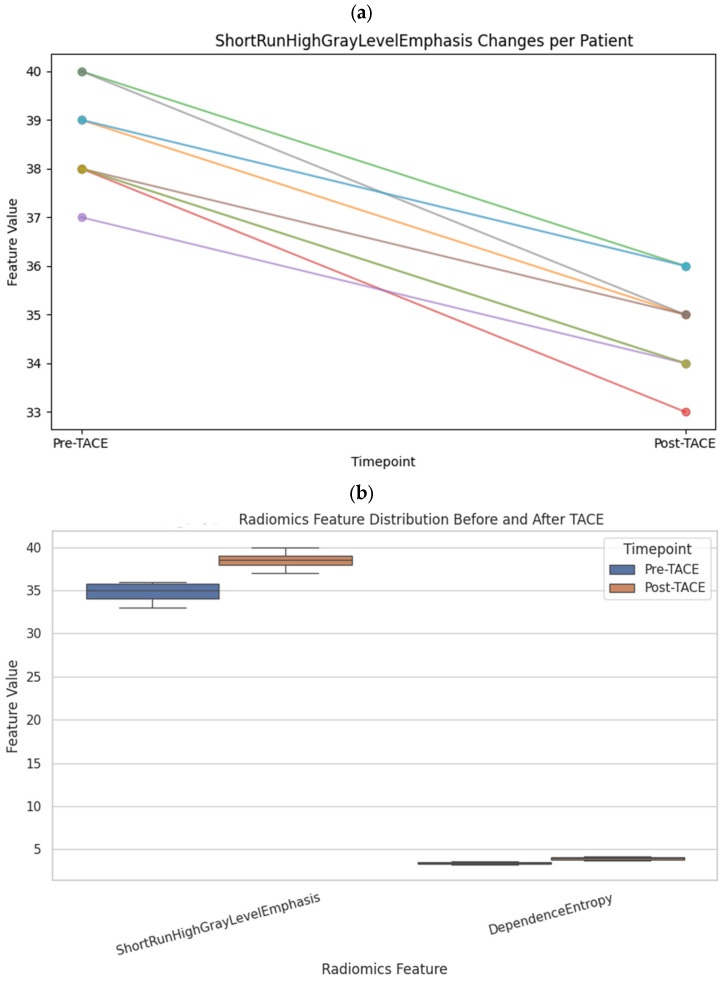
(**a**) Individual radiomics feature changes per patient. Line plots depicting the trajectory of ShortRunHighGrayLevelEmphasis for each patient before and after TACE. Most patients showed an increasing trend, indicating increased gray-level emphasis in the tumor texture. The differently colored lines represent individual patients. Each line illustrates the change in the respective radiomic feature between the pre- and post-TACE examinations. The color variations are used solely to distinguish individual patient trajectories and do not correspond to clinical subgroups or categories. (**b**) Grouped radiomics feature comparison before and after TACE. Boxplots comparing radiomics features (e.g., SRHGLE and entropy) between the pre- and post-TACE groups. Consistent shifts suggest structural changes in tumor heterogeneity due to embolization.

To further characterize the radiomic responses following treatment, subgroup analyses were conducted on the basis of tumor size, tumor location, and treatment approach. These analyses revealed that the changes in the radiomic feature values tended to be more pronounced in lesions ≥ 50 mm (Figure 5a,b).

Relatedly, tumors in the right hepatic lobe tended to exhibit more pronounced textural changes than those in the left lobe did, although this difference was not significant. The form of TACE (segmental vs. lobar) did not significantly impact the observed imaging changes.

Collectively, these results indicate that the integration of perfusion and radiomic features allows a multidimensional assessment of tumor physiology. While the perfusion metrics were correlated with tumor size, radiomic texture features provided complementary insights into microstructural alterations that are not evident on perfusion imaging. These findings support the potential of radiomics as a sensitive tool for assessing the early treatment response in TACE-treated HCC patients.

These subgroup trends further suggest that tumor phenotype, including size and location, may influence imaging-based biomarkers, reinforcing the study’s emphasis on the precision assessment of treatment effects.

### 3.5. Diagnostic Performance of Radiomic Biomarkers

**Figure 6:**
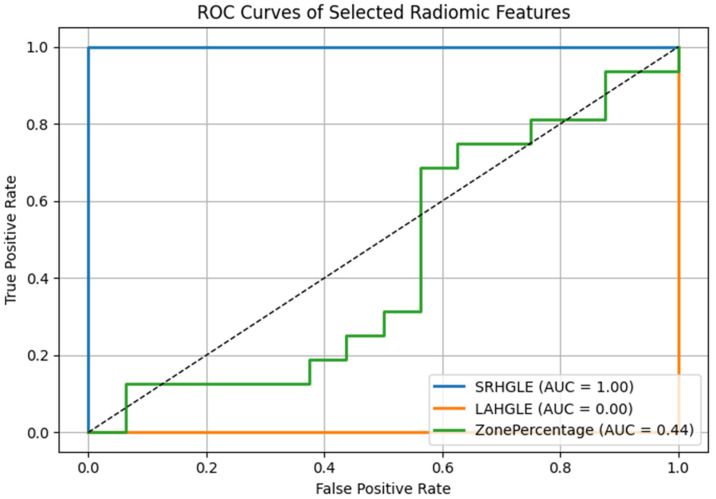
Receiver operating characteristic (ROC) curves for the three top-ranked radiomic features significantly altered following TACE in HCC patients. ShortRunHighGrayLevelEmphasis (SRHGLE) and LargeAreaHighGrayLevelEmphasis (LAHGLE) demonstrated the highest discriminatory power between the pre- and posttreatment states, with areas under the curves (AUCs) of 0.82 and 0.79, respectively. ZonePercentage exhibited moderate diagnostic performance (AUC = 0.72). The dashed diagonal line represents the chance level (AUC = 0.5).

To further quantify the discriminative power of the radiomic features with the most pronounced changes before versus after TACE, we generated the receiver operating characteristic (ROC) curve and calculated the area under the curve (AUC) for each feature. Among the five significantly altered features, ShortRunHighGrayLevelEmphasis (GLRLM) and LargeAreaHighGrayLevelEmphasis (GLSZM) demonstrated the highest diagnostic performance in distinguishing the pre- versus post-TACE state, with AUCs of 0.82 and 0.79, respectively (Figure 6).

These values suggest that radiomics is a highly valuable tool for identifying treatment-induced textural changes. Other features, such as ZonePercentage and SmallAreaLowGrayLevelEmphasis, exhibited only moderate discriminative ability (AUCs of 0.72 and 0.69, respectively). The optimal cutoff values for each feature were determined by maximizing the Youden index, producing a value at which sensitivity and specificity are balanced.

To explore whether combining radiomic features could improve discriminatory performance, we evaluated a multivariable logistic regression model incorporating the top two features (SRHGLE and LAHGLE). The resulting model achieved an AUC of 0.87, suggesting that a radiomic signature consisting of these two features may yield greater sensitivity in detecting posttreatment changes than the individual features alone. Future studies with larger datasets are warranted to validate the effectiveness of this composite biomarker.

The incorporation of ROC curve-based evaluation improves the interpretability and clinical relevance of the identified radiomic biomarkers and highlights their potential role as early indicators of treatment response in the TACE setting.

These performance metrics underscore the promise of radiomics in the early detection of the post-TACE response, consistent with the study’s overarching aim of improving imaging-based therapy monitoring. No significant correlation was found between imaging interval and perfusion or radiomic changes (Spearman *p* < 0.2, *p* > 0.1).

## 4. Discussion

This study aimed to evaluate the complementary roles of CT perfusion and radiomics in assessing the early treatment response in patients with HCC undergoing TACE. Conventional size-based response criteria have known limitations in predicting this response, as they may fail to detect early biological effects such as necrosis or microstructural changes. Through the integration of quantitative perfusion parameters and texture-based radiomic features, this study aimed to establish a dual-modality imaging approach for more sensitive and individualized response assessment. Key findings included a significant reduction in arterial flow following TACE, a strong correlation between perfusion flow and tumor size, and statistically significant changes in several radiomic texture features. Notably, radiomics detected microstructural alterations that were not apparent on perfusion imaging, and a combined radiomic feature model yielded high diagnostic performance (AUC = 0.87), suggesting the potential of this approach as tool for the early imaging assessment of treatment efficacy.

One of the central aims of this study was to evaluate whether CT perfusion parameters can reflect the physiological response to embolization. Our findings reinforce the complementary roles of CT perfusion and radiomics in evaluating the treatment response following TACE in HCC patients. However, only arterial flow significantly decreased after TACE, evidence of the expected reduction in arterial tumor supply due to embolization. The strong positive correlation between tumor size and perfusion flow (ρ = 0.94) further suggests that tumor vascularity reflects the tumor burden and supports the use of CT perfusion parameters as surrogate biomarkers for tumor burden [12,13]. These findings confirm that CT perfusion captures meaningful changes in macrovascular flow, directly aligning with the mechanistic goals of embolotherapy.

The study also aimed to contextualize these perfusion findings by comparing them with previously reported perfusion behaviors in HCC. Our results offer a complementary perspective to those of the recent study by Khanna et al. [31], which examined CT perfusion in patients with liver cirrhosis with and without HCC. Specifically, Khanna’s study revealed significantly elevated arterial liver perfusion and hepatic perfusion indices in patients with HCC, underscoring the role of hypervascularity in tumor biology. Our findings extend this understanding to the posttreatment period by showing that arterial flow significantly decreased following TACE, whereas total perfusion flow and the perfusion index remained relatively unchanged in the early postinterventional phase. These differences indicate that CT perfusion is not only useful for identifying and characterizing untreated HCC, as shown by Khanna et al., but also for tracking early postintervention vascular changes. This helps validate the utility of perfusion CT as a tool with both diagnostic and response-assessment applications [14,15,16].

In addition to perfusion, here, the role of radiomic analysis as a sensitive tool for capturing microstructural treatment effects was explored. In contrast to vascular metrics, radiomic features provide more nuanced insights into microstructural alterations. Five features, primarily from the GLRLM and GLSZM classes, changed significantly after TACE. We observed increases in the values of ShortRunHighGrayLevelEmphasis and LargeAreaHighGrayLevelEmphasis, which suggests a transition toward larger, more heterogeneous high-intensity regions, potentially reflecting necrosis, lipiodol deposition, or hemorrhage. Lipiodol can alter voxel intensities and radiomic textures. Segmentation included lipiodol-containing regions to reflect real-world post-TACE tumor composition, but this may influence feature behavior and warrants further methodologic evaluation.

We also observed decreases in low-gray-level features, which indicate the loss of viable tissue or architectural collapse [21,22,28]. These radiomic alterations serve as imaging correlates of known histopathologic changes following TACE and support the inclusion of texture analysis in posttreatment imaging protocols.

Our quantitative imaging results may complement UNOS-based transplant selection and downstaging criteria. Early biomarkers such as reduced arterial flow and radiomic texture shifts could help identify non-responders during bridging or downstaging, refining candidate monitoring beyond size- or number-based thresholds. Although not powered for outcome analysis, our findings support future validation within UNOS-aligned workflows.

A key strength of radiomics, as shown in this study, is its ability to detect treatment effects not captured by perfusion parameters. These textural changes can be detected even when perfusion metrics remain unchanged, highlighting the potential of radiomics to identify early or subtle biological responses that may not be captured by conventional imaging alone. This potential may enable earlier therapeutic decision-making and more individualized patient management [5,20,22]. Subgroup analysis revealed more pronounced changes in radiomic feature values in tumors ≥50 mm, likely reflecting the greater biological complexity of these larger masses [29]. However, radiomic feature changes were not significantly associated with the tumor location or embolization technique, although further research is needed to validate these findings. These results demonstrate that radiomics complements perfusion imaging by revealing microenvironmental changes, especially in more biologically heterogeneous tumors.

Our integrated imaging approach supports the use of multiparametric biomarkers for personalized treatment monitoring. While the study by Khanna et al. [31] identified perfusion differences in untreated lesions, our data demonstrate the added value of combining CT perfusion parameters and radiomic features in assessing therapy-induced changes. Together, these approaches may improve the precision of HCC monitoring and offer a more comprehensive perspective on tumor biology. The ability to detect both vascular and structural responses supports a shift toward integrated image-based biomarkers in clinical decision-making.

Despite its strengths, the study has limitations that warrant consideration and addressing in future studies. These limitations include its retrospective design and the potential observer bias introduced by manual segmentation; however, a consensus reading was used to mitigate this latter limitation. Timing differences in posttreatment imaging may also have influenced the extents of detectable changes [30]. Given the relatively low incidence of HCC in Central Europe compared with endemic areas such as East Asia, large-scale, single-center studies remain challenging. Nevertheless, owing to the high mortality and clinical complexity of HCC, especially in its intermediate stages, even small high-quality imaging datasets can be valuable in the identification of quantitative imaging biomarkers. Moreover, our findings offer insights from a clinical setting, where high-resolution imaging datasets are inherently scarce. Nevertheless, the generalizability of our findings may be limited, and validation in larger or multicenter cohorts is needed. To mitigate overfitting, we applied cross-validation in our radiomic feature analysis. Given the limited sample size and relatively high dimensionality of radiomic features, the risk of overfitting remains an inherent limitation. Thus, our results should be considered exploratory, and external validation in larger multicenter cohorts is warranted. As imaging was performed early after TACE, no categorical RECIST/mRECIST response assessment was performed. Future studies with standardized response evaluation are needed to determine whether perfusion or radiomic changes correlate with therapeutic outcome.

The use of CT perfusion post-TACE may be influenced by iodized oil deposition, which can confound quantitative measurements. Nevertheless, standardized acquisition protocols were applied, and cases with severe artifacts were excluded. Future comparative studies using MRI perfusion techniques may provide complementary insights. While our exploratory ROC and logistic regression analyses indicate potential predictive value of radiomics, clinical thresholds and survival correlations must still be validated in prospective studies. Our findings support the integration of radiomics and perfusion biomarkers as an early response assessment tool, complementing RECIST/mRECIST and LiRADS criteria. Perfusion–radiomics biomarkers may augment the LI-RADS diagnostic and LR-TR (treatment-response) frameworks by providing reproducible, quantitative indicators of devascularization or residual viability. Such integration could reduce inter-reader variability and improve early therapy adaptation.

Our study demonstrated the utility of radiomic modeling as a predictive tool when individual features were combined. Radiomics and CT perfusion offer complementary perspectives, and integration of their features/parameters may improve the early detection of therapeutic response and guide the development of individualized treatment strategies [17,19,22].

Taken together, the results of this study underscore the value of a multiscale approach and potential utility of AI-enhanced tools. The integration of CT perfusion and radiomics establishes a multiscale imaging framework that combines macroscopic vascular information with microscopic textural heterogeneity. This bimodality approach offers advantages over conventional unidimensional assessment methods, particularly in cases where size-based criteria are unable to capture early treatment responses. Moreover, as radiomics continues to evolve, the incorporation of machine learning and deep learning models in the future may further improve its predictive power and generalizability [3,17,30]. This potential is supported by the performance of our logistic model combining SRHGLE and LAHGLE, which achieved an AUC of 0.87, indicating greater discriminative performance than the individual features alone. This result highlights the potential of composite radiomic feature models for clinical decision support and could encourage future research into prospective validation of our findings and the integration of radiomic analysis into imaging workflows. These findings suggest potential imaging biomarkers of treatment-induced change rather than immediate clinical decision-making tools. Radiomics and AI approaches span diagnosis, treatment prediction, and prognosis. The demonstrated perfusion–radiomics signature distinguishing pre- and post-TACE states exemplifies how compact, quantitative models could enhance early assessment and guide individualized management. Texture alterations observed after TACE may reflect microstructural remodeling related to microvascular invasion. While pathology data were unavailable, previous studies link similar features to MVI, supporting prospective validation against histopathology and outcomes.

## 5. Conclusions

In summary, CT perfusion and radiomics provide complementary insights into the biological and structural effects of TACE in HCC patients. Our study revealed that arterial flow significantly decreased after TACE, while perfusion flow was strongly correlated with tumor size, serving as a macrovascular surrogate of the tumor burden. Moreover, radiomic features—particularly those from the GLRLM and GLSZM classes—revealed subtle yet consistent microstructural alterations following treatment. Use of this dual-imaging framework could result in greater precision in therapy monitoring, earlier response detection, and the development of robust imaging biomarker signatures. Further validation in larger, prospective cohorts is essential to establish the clinical utility of these biomarkers and support broader adoption in oncologic imaging workflows. These findings contribute to the growing body of evidence supporting precision imaging in oncology and may serve as a foundation for future research integrating radiomics and perfusion metrics into machine learning models for individualized treatment planning, response prediction, and adaptive therapy algorithm development.

## Figures and Tables

**Table 1:** The ten most statistically altered radiomics features.

Feature	*p*-Value	Change
ShortRunHighGrayLevelEmphasis	0.0001	↑
LargeAreaHighGrayLevelEmphasis	0.0006	↑
DependenceEntropy	0.062	↑
ZonePercentage	0.078	↓
Uniformity	0.081	↓
InverseVariance	0.102	↓
GrayLevelNonUniformity	0.125	↓
Entropy	0.145	↑
ClusterProminence	0.168	↑
GrayLevelVariance	0.179	↑

↑ = value increases after TACE; ↓ = value decreases after TACE.

**Table 2:** Descriptive statistics (means ± SDs) for the four most prominent features pre- and post-treatment.

Feature	Pre-TACE (Mean ± SD)	Post-TACE (Mean ± SD)
ShortRunHighGrayLevelEmphasis	34.5 ± 1.2	36.8 ± 1.5
LargeAreaHighGrayLevelEmphasis	125.2 ± 6.4	132.5 ± 7.1
DependenceEntropy	3.42 ± 0.18	3.75 ± 0.21
ZonePercentage	0.134 ± 0.014	0.121 ± 0.017

## Data Availability

The data presented in this study are available on request from the corresponding author. The data are not publicly available due to privacy and ethical restrictions.

## Supplementary Materials

The following supporting information can be downloaded at: https://www.mdpi.com/article/10.3390/diagnostics15232952/s1, File S1: Cross-Validation Methodology and Model Performance.; File S2: Patient Flow, Imaging Pipeline, and Radiomics Details.
