# Supplementary material for: Quantitative CT Perfusion and Radiomics Reveal Complementary Markers of Treatment Response in HCC Patients Undergoing TACE
Source: Diagnostics (Basel). 2025 Nov 21;15(23):2952. doi: 10.3390/diagnostics15232952 (PMC12691162; doi:10.3390/diagnostics15232952)
Supplement: Supplementary file 1 [file diagnostics-15-02952-s001.zip › Supplementary_File_S1.pdf]

Supplementary File S1 – Cross-Validation Methodology and Model Performance

Study Objective & Context

This supplementary file provides methodological details for the radiomics model development, validation,
and performance assessment in the study evaluating CT perfusion and radiomics biomarkers in HCC patients treated with TACE.
Given the exploratory nature and limited sample size (n=32), the primary goal was to mitigate overfitting and ensure reproducibility.

Cross-Validation Design

A repeated k-fold cross-validation strategy was used:
• Method: 5-fold cross-validation, repeated 5 times
• Total models trained: 25
• Stratification: by pre/post-TACE label
• Performance averaged across folds

Feature Preprocessing

Radiomic preprocessing followed IBSI standards including voxel resampling (1x1x1 mm), z-score normalization, and fixed bin-width discretization (bin width = 25).

Feature Selection

To reduce dimensionality and multicollinearity:
• Variance threshold applied to remove near-constant features
• Pairwise Spearman correlation filtering ($|\rho| > 0.9 \rightarrow$ one feature retained)
• Selection based on statistical significance and biological interpretability

Modeling Pipeline

The final logistic regression model included the top two radiomic features (SRHGLE and LAHGLE).
A fixed random seed ensured reproducibility. No hyperparameter tuning was applied to avoid overfitting.

Performance Metrics

The following metrics were recorded for each CV iteration:
• AUC (primary)
• Accuracy, sensitivity, specificity
• 95% confidence intervals via bootstrap
• Calibration assessment (calibration curve)

Reproducibility Notes
Random state was set to 42 for all folds and repetitions.
Implementation was performed in Python (v3.11) using scikit-learn and Statsmodels.

Performance Metric Definitions:

Metric	Definition
AUC	Area under receiver operating characteristic curve
Accuracy	Correct predictions / total cases
Sensitivity	True positive rate
Specificity	True negative rate
Calibration	Visual evaluation of predicted vs observed probabilities

.

Model Performance Summary
The following table summarizes cross-validated performance metrics for the two-feature logistic regression classifier (SRHGLE + LAHGLE). Values represent mean performance across 5×5 repeated cross-validation with 95% bootstrapped confidence intervals.

Metric	Mean (CV)	95% CI
**AUC**	0.87	0.78–0.94
**Accuracy**	0.81	0.71–0.89
**Sensitivity**	0.83	0.72–0.91
**Specificity**	0.80	0.70–0.88

Model Output Clarification: The model discriminated pre-TACE vs post-TACE state, capturing treatment-induced texture phenotype changes. Evaluation of clinical outcome prediction is planned for future prospective studies.

Reproducible Modeling Pseudocode

```
for repeat in range(5):
    kf = KFold(n_splits=5, shuffle=True, random_state=42)
    for train_index, test_index in kf.split(data):
```

```
        # Split data
        X_train, X_test = X[train_index], X[test_index]
        y_train, y_test = y[train_index], y[test_index]

        # Train logistic regression
        model = LogisticRegression(max_iter=1000, random_state=42)
        model.fit(X_train, y_train)

        # Evaluate performance
        y_pred = model.predict_proba(X_test)[:,1]
        auc = roc_auc_score(y_test, y_pred)
        store_metrics(auc, other_metrics...)

# Report mean and 95% CI
```
