# Supplementary material for: Quantitative CT Perfusion and Radiomics Reveal Complementary Markers of Treatment Response in HCC Patients Undergoing TACE
Source: Diagnostics (Basel). 2025 Nov 21;15(23):2952. doi: 10.3390/diagnostics15232952 (PMC12691162; doi:10.3390/diagnostics15232952)
Supplement: Supplementary file 1 [file diagnostics-15-02952-s001.zip › Supplementary_File_S2.pdf]

Supplementary File S2 – Patient Flow, Imaging Pipeline, and Radiomics Details

1. Patient Inclusion Flow (STROBE Diagram)

A total cohort of hepatocellular carcinoma (HCC) patients undergoing TACE was screened.
Cases were excluded if pre- or post-TACE CT perfusion was unavailable or if severe lipiodol-related artifacts impaired tumor visualization or segmentation.
The final study population consisted of 32 patients.

HCC patients screened	68
Excluded: missing pre/post CT perfusion	22
Excluded: severe lipiodol artifacts	3
Excluded: lost to follow-up / incomplete data	11
Final included patients	32

2. CT Acquisition & Preprocessing

All CT scans were performed using multiphase liver imaging protocols.
Preprocessing followed Image Biomarker Standardization Initiative (IBSI) recommendations:

- Isotropic resampling: 1 × 1 × 1 mm
- Z-score intensity normalization
- Fixed bin-width intensity discretization (bin width = 25 HU)
- Harmonized reconstruction kernel
- Perfusion maps generated using standardized vendor software

3. Tumor Segmentation

Whole-tumor 3D segmentation was performed on arterial-phase CT and perfusion maps.

• Manual segmentation by two radiologists in consensus
• Whole-lesion volume including necrotic and lipiodol regions
• Severe lipiodol artifacts were excluded
• Segmentation aimed to capture full post-TACE tumor phenotype

4. Radiomics Features Extracted (Template)

A total of 98 radiomic features were extracted from the segmented volume.
Below is a structured template of feature groups.

Feature Class	Examples
First-order statistics	Mean, Variance, Skewness, Kurtosis
GLCM	Contrast, Correlation, Homogeneity
GLRLM	Short-Run High Gray Level Emphasis (SRHGLE), Long-Run Emphasis
GLSZM	Large-Area High Gray Level Emphasis (LAHGLE), Zone Variance
Shape	Volume, Surface-Area-to-Volume ratio
NGTDM / GLDM	Coarseness, Dependence Variance

5. Scan Interval Effect Analysis

The median interval between pre- and post-TACE CT was 30 days.
No significant correlation was observed between scan interval and radiomic or perfusion feature changes
(Spearman $|\rho| < 0.2$, $p > 0.1$).
